# The Role of Vitamin K in Cholestatic Liver Disease

**DOI:** 10.3390/nu13082515

**Published:** 2021-07-23

**Authors:** Halima Sultana, Michio Komai, Hitoshi Shirakawa

**Affiliations:** 1 Laboratory of Nutrition, Graduate School of Agricultural Science, Tohoku University, 468-1 Aramaki Aza Aoba, Aoba-ku, Sendai 980-8572, Japan; sultana.halima.d4@tohoku.ac.jp (H.S.); mkomai@m.tohoku.ac.jp (M.K.); 2 International Education and Research Center for Food Agricultural Immunology, Graduate School of Agricultural Science, Tohoku University, 468-1 Aramaki Aza Aoba, Aoba-ku, Sendai 980-8572, Japan

**Keywords:** vitamin K, pregnane X receptor, bile acid metabolism, cholestasis

## Abstract

Vitamin K (VK) is a ligand of the pregnane X receptor (PXR), which plays a critical role in the detoxification of xenobiotics and metabolism of bile acids. VK_1_ may reduce the risk of death in patients with chronic liver failure. VK deficiency is associated with intrahepatic cholestasis, and is already being used as a drug for cholestasis-induced liver fibrosis in China. In Japan, to treat osteoporosis in patients with primary biliary cholangitis, VK_2_ formulations are prescribed, along with vitamin D_3_. Animal studies have revealed that after bile duct ligation-induced cholestasis, PXR knockout mice manifested more hepatic damage than wild-type mice. Ligand-mediated activation of PXR improves biochemical parameters. Rifampicin is a well-known human PXR ligand that has been used to treat intractable pruritus in severe cholestasis. In addition to its anti-cholestatic properties, PXR has anti-fibrotic and anti-inflammatory effects. However, because of the scarcity of animal studies, the mechanism of the effect of VK on cholestasis-related liver disease has not yet been revealed. Moreover, the application of VK in cholestasis-related diseases is controversial. Considering this background, the present review focuses on the effect of VK in cholestasis-related diseases, emphasizing its function as a modulator of PXR.

## 1. Vitamin K

Vitamin K (VK) is a fat-soluble vitamin that acts as a cofactor of γ-glutamyl carboxylase (GGCX). VK is important in blood coagulation and bone formation. GGCX is required for the post-translational modification of several precursor proteins by γ-glutamyl carboxylation in multiple tissues. It catalyzes the addition of a carboxy group to glutamate residues in VK-dependent (VKD) substrate proteins. This reaction is coupled by the oxidization of VK hydroquinone to VK epoxide. Several glutamate residues are required to be γ-carboxylated for the activation of VKD proteins. The modified glutamate residue is named Gla residue. Cyclic use of VK is necessary for its continued function as a cofactor for GGCX [1]. For recycling, VK epoxide is reduced by VK epoxide reductase (VKOR) [2]. Gla residues allow the activation of coagulation factors and calcium binding to Gla proteins, such as prothrombin, factor VII, factor IX, factor X, protein C, protein S, and protein Z [2].

Beyond blood and bone homeostasis, VK is also involved in many physiological and biological processes that include inflammation, testosterone production, cancer progression, a neuroprotective effect, bile acid (BA) metabolism, insulin secretion, and type 2 diabetes [3,4,5,6,7,8,9]. Deficiency of VK may be associated with many pathological conditions, including weakness, osteoporosis, osteoarthritis, cognitive impairment, and coronary artery disease [10,11,12]. Most of these are age-related diseases that impose considerable economic burdens on social security systems. To overcome this challenge, novel and efficient nutritional options are urgently needed. Many studies have shown the beneficial effects of VK with no toxicity or adverse effects related to high-dose treatment. Thus, naturally occurring VK could be a potential dietary supplement for many of the aforementioned diseases.

VK exists naturally in two bioactive forms, i.e., phylloquinone (VK_1_) and menaquinones (VK_2_ or MK-n). Humans consume VK_1_ mostly from vegetable oils and green leafy vegetables, such as kale, spinach, and broccoli. Nevertheless, menaquinones are abundant in fermented products and animal-based products. Fermented soybean products, such as natto, and fermented milk-based products, such as cheese and soured milk, contain an adequate amount of menaquinone-7 (MK-7) and other MK-n. Animal organs, meat, fish, and eggs are enriched with MK-4. Of the total intake of VK, approximately 10% of menaquinones are stored in the liver [13].

Thijssen reported that VK_1_ is stored in all tissues in humans. A relatively high level of VK_1_ can be found in the liver, heart, and pancreas, and low levels can be found in the brain, lungs, and kidney [14]. However, VK_2_ is stored in most tissues, with relatively high levels in the brain and kidneys [14]. We previously reported that orally administered VK_1_ is distributed to most of the tissues, and is efficiently converted to MK-4 in the brain, testis, kidney, and spleen of Wistar rats. This study also showed that an abundance of MK-4 is distributed and stored in various tissues in VK-deficient rats after the oral administration of VK_1_ [15].

There are four main modes of VK action. The classical mechanism of VK as a cofactor for GGCX was revealed in 1974 [16,17]. This reaction requires the reduced form of VK (hydroquinone form) generated by quinone oxidoreductase or VK epoxide reductase, which creates a VK cycle for reuse. Both VK_1_ and K_2_ operate in this mode of action. In 2003, another mode of function was revealed when it was reported that MK-4 functions as a ligand of PXR [3]. Upon MK-4 binding, PXR forms a heterodimer with a retinoid X receptor. This complex binds to PXR-responsive elements within the regulatory regions of target genes [18]. In 2006, we reported an important anti-inflammatory mode of action of VK [19]. In this mode of action, VK suppresses inflammation by inactivating the nuclear factor kappa B (NF-κB) signaling pathway [4,20]. Another function of MK-4 as an activator of protein kinase A (PKA) was recently reported [2]. A typical substrate of PKA is the cyclic AMP responsive element-binding protein (CREB), which binds to cyclic AMP responsive elements within the enhancer or promoter regions of target genes when CREB is phosphorylated [21].

## 2. Pregnane X Receptor

PXR (NR1I2, also termed SXR) is now considered a master regulator in the field of toxicology. PXR was identified in 1998 as a member of the nuclear receptor (NR) superfamily of ligand-activated transcription factors. The liver and intestine are the major organs where detoxification occurs. PXR is predominantly expressed in these organs, and, to a lesser extent, in the kidney [18,22,23]. The expression of PXR is low in other tissues that include the lung, stomach, uterus, ovary, breast, adrenal gland, bone marrow, and some parts of the brain [24].

The reactions of drug/xenobiotic metabolism can be divided into three phases: phase I (hydroxylation), phase II (conjugation), and phase III (transport). Numerous genes involved in drug/xenobiotic metabolism are regulated by PXR [25]. In general, PXR is activated by xenobiotics, such as antibiotics, pharmacological and herbal compounds, dietary substances, and exogenous and endogenous substances, such as BAs and their precursors. PXR activation, in turn, is important in the regulation of many drug-metabolizing enzymes and drug transporters [26,27,28,29,30]. Enzymes of the CYP3A subfamily are particularly important, because they are involved in the metabolism of around 50% of prescribed drugs [31,32]. Recently, several studies have revealed the importance of PXR in diverse physiological functions, such as inflammation, bone homeostasis, lipid and BA homeostasis, vitamin D (VD) metabolism, and energy homeostasis, as well as in many diseases, such as cholestasis, inflammatory bowel disorders, and cancer [29].

Human PXR is the product of the nuclear receptor subfamily 1 group I member 2 (*NR1I2*) gene. The gene is located on chromosome 3, and contains 10 exons separated by nine introns. Like other NRs, PXR has an N-terminal domain, a DNA-binding domain (DBD), a hinge region, and a ligand-binding domain (LBD) [24]. However, although NRs generally interact selectively with their physiological ligands, the enlarged, flexible, hydrophobic LBD of PXR allows it to be activated by an enormous variety of substances. PXR LBD contains an insert of approximately 60 residues that is not present in other NRs [33]. Because of these special structural features, PXR LBD can change its shape to accommodate miscellaneous ligands depending on their nature [26]. Human and rodent PXR share > 94% amino acid sequence identity in the DBD, but only 76–82% amino acid sequence identity in LBD [34].

The binding of a potential ligand with PXR causes the dissociation of corepressors. This stimulates the association of the coactivators, resulting in the activation of transcription [35]. Coactivator recruitment plays a vital role in fixing the ligand properly in the large LBD cavity after the release of the corepressor [24]. Species-specific ligand preference by PXR constitutes a considerable challenge for studies of PXR function in animals. For example, pregnane 16α-carbonitrile (PCN) is a synthetic, well-tolerated steroidal anti-glucocorticoid that alters drug responses by inducing hepatic microsomal drug-metabolizing enzymes in animals and humans. PCN is a substantially stronger activator of rat or mouse PXR than human or rabbit PXR. Similarly, rifampicin (Rif), an antibiotic and well-known anti-tuberculosis drug, is a strong activator of human or rabbit PXR, but a very weak activator of mouse or rat PXR [36]. This species-specific preference limits the relevance of evaluations of the toxicity and functionality of PXR ligands in rodents to human physiology. To overcome this issue, a number of mouse models with humanized PXR based on different strategies have been developed [37,38,39,40].

## 3. Vitamin K and Pregnane X Receptor

In 2003, Tabb et al. reported for the first time that MK-4 directly acts as a ligand of PXR and, upon binding, transcriptionally activates PXR, which ultimately promotes the association of coactivators with PXR. In turn, activated PXR plays an important role in regulating the gene expression involved in bone homeostasis [3]. Later, Ichikawa et al. further evaluated the effect of MK-4 mediated PXR activation in bone homeostasis by analyzing the alteration of mRNA expression by Rif and MK-4 [41]. This study showed that the activation of PXR by MK-4 regulates the transcription of extracellular matrix-related genes and cell surface markers, which are involved in both osteoblastogenesis and osteoclastogenesis [41]. The PXR-mediated effect of VK was also subsequently observed in human hepatocellular carcinoma cells [42]. This study demonstrated that the activation of PXR by MK-4 suppresses proliferation and motility, which plays a significant role in intrahepatic metastasis of hepatocellular carcinoma cells, thereby preventing the occurrence and recurrence of these cells by acting as a cofactor of GGCX, as well as a ligand to enhance the activation of PXR. In 2015, another group of researchers showed that a combination of MK-4 and lithocholic acid (LCA), a secondary BA produced by intestinal microbiota, can activate PXR synergistically, resulting in the subsequent expression of typical PXR target genes *CYP3A4* and *CYP2C9* during the fetal hepatocyte stage [43]. The authors demonstrated that LCA and MK-4 could drive the metabolic maturation of human embryonic stem cell-derived hepatocytes [43].

Studies have been conducted to show the role of VK on cholestatic liver disease. The role of PXR in bile metabolism has also been studied. However, to the best of our knowledge, no studies or reviews have shown the potential role of VK as a modulator of PXR in cholestatic liver diseases. In the present review, we have discussed the effect of VK in cholestasis-related liver diseases, emphasizing its function as a modulator of PXR. We have searched the literature by using keywords related to the present review, using Scopus, NCBI, and a general internet search, and then selected the relevant articles. We looked through the reference lists of the selected articles for other relevant articles, books, and book chapters as well.

## 4. Overview of Bile Acids Metabolism

**Figure 1:**
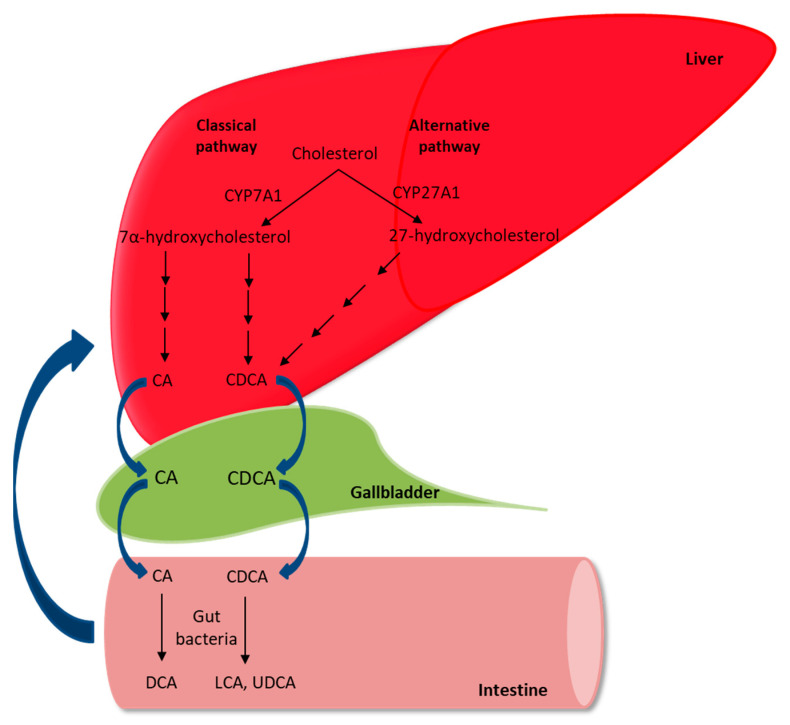
A simplified view of bile acid metabolism in humans. CYP7A1, cholesterol 7α-hydroxylase; CYP27A1, sterol-27 hydroxylase; CA, cholic acid; CDCA, chenodeoxycholic acid; MCA, muricholic acid; DCA, deoxycholic acid; LCA, lithocholic acid; and UDCA, ursodeoxycholic acid.

For a better understanding of cholestatic liver disease, the metabolism of BAs is discussed here in brief. BAs are amphipathic sterols that are synthesized from cholesterol in the liver, stored in the gallbladder, and secreted into the intestine following food intake. BAs act as physiological detergents, which are required for intestinal transport and absorption of dietary lipids, including fat-soluble vitamins [44]. There are two pathways for the synthesis of BAs: the classic or neutral pathway and the alternative or acidic pathway. The classic pathway is the predominant pathway initiated by cholesterol 7α-hydroxylase (CYP7A1). Cholesterol is converted into two primary BAs in the human liver, i.e., chenodeoxycholic acid (CDCA) and cholic acid (CA). The distribution of these two BAs is determined by the activity of sterol 12-α-hydroxylase (CYP8B1). Subsequently, these BAs are conjugated primarily with glycine and taurine in humans, transported to the gallbladder through the bile canaliculi, and stored along with cholesterol and phospholipids. Following food intake, the gallbladder extricates BAs into the intestine, where they help in the absorption of lipids and fat-soluble vitamins. Primary BAs are converted into secondary BAs by the gut microbiota after deconjugation and dehydroxylation. In the intestine, unconjugated BAs passively diffuse into enterocytes, and the active uptake of conjugated BAs occurs generally in the ileum by the apical sodium-dependent bile acid transporter (ASBT). Approximately 95% of BAs are reabsorbed into enterocytes, and 5% are excreted via feces. CA, CDCA, deoxycholic acid (DCA), and a small portion of LCA are transported back to the liver via the portal vein through specific transporters in the apical and basolateral membranes of enterocytes, thereby inhibiting BA synthesis [44] (Figure 1).

## 5. Cholestatic Liver Disease

Cholestasis is associated with impaired bile formation by hepatocytes or impaired bile secretion and flow at the level of cholangiocytes by cholelithiasis or tumor [45]. Cholestasis can be either extrahepatic or intrahepatic. The extrahepatic form is caused by choledocholithiasis, stones, tumors, and parasitic infections. The intrahepatic form is caused by immune-mediated conditions; exposure to medications that include steroids, nonsteroidal anti-inflammatory drugs or antibiotics, and anti-diabetic agents; and by inborn errors of cholesterol or BA biosynthesis and metabolism. Cholestasis causes the accumulation of potentially toxic BAs and bile salts in the systemic circulation and intestine. Hence, cholestasis itself causes bile duct injury, resulting in further accumulation of toxic BAs, which cause further damage to the bile duct [46]. Moreover, it is a major complication that profoundly affects the success rate of liver transplantation [47]. Conventionally, cholestasis that persists for more than six months is considered chronic [48]. The most frequent chronic cholestatic liver diseases are primary biliary cholangitis (PBC) and primary sclerosing cholangitis (PSC). Both can be considered model diseases concerning the management of cholestasis [46]. PBC is characterized by the immune-mediated destruction of epithelial cells of the intrahepatic bile ducts. PSC is a chronic immune-mediated disease of the larger intra- and extrahepatic bile ducts, which results in persistent cholestasis [49]. Common clinical manifestations of cholestatic liver disease include fatigue, pruritus, and jaundice. Osteoporosis is also frequently observed in PBC [50]. Early biochemical markers of cholestasis include an elevated level of serum alkaline phosphatase and γ-glutamyltranspeptidase, followed by conjugated hyperbilirubinemia at more advanced stages [48]. The major abnormalities of cholestatic patients are an elevated level of circulating primary BAs and increased formation of sulfate-conjugated BAs. Renal excretion is the major method of BA elimination in patients with severe cholestasis [51]. In advanced cholestasis, the ratio of primary BAs (CA/CDCA) increases in the serum, and the proportion of unconjugated BAs, as well as concentrations of the secondary BA (DCA), is reduced [52]. The physiological consequences of reduced intestinal BAs cause maldigestion of triacylglycerol and malabsorption of fat-soluble vitamins. The pathophysiological level of BAs induces inflammation [53]. If untreated, increased circulating BAs cause pruritus, and can eventually cause apoptosis or necrosis of hepatocytes, leading to progressive hepatic fibrosis and even cirrhosis that can cause death due to hepatic failure or the complications of portal hypertension [52,54,55].

## 6. Vitamin K Deficiency in Cholestatic Liver Disease

The biological significance of VK in the regulation of BA synthesis is unclear. However, VK deficiency is commonly observed in cholestasis [56,57,58,59,60]. VK deficiency is usually diagnosed by measuring prothrombin time (PT), which is prolonged in different forms of liver disease [60]. Kowdley et al. showed that a lower level of VK_1_ is common in patients with PBC, and it is associated with decreased serum levels of vitamins A and E [59]. VK deficiency is reportedly prevalent in children with mild to moderate chronic cholestatic liver disease, and it was demonstrated that VK deficiency was significantly related to the level of cholestasis and severity of liver disease in children, whereas children without cholestasis did not have a VK deficiency [60]. The international normalized ratio (INR) is a marker used to determine whether coagulopathy reversal is necessary. Strople et al. demonstrated that all cholestatic adults and children with elevated INR were VK deficient [57]. This deficit was not even corrected by oral consumption of VK, because intestinal absorption is compromised in cholestasis. VK deficiency was also associated with intrahepatic cholestasis during pregnancy [56]. Low levels of VK may cause dysregulation of BA synthesis, leading to the upregulation of *CYP7A1* and *CYP8B1* expression levels [56]. However, few studies have assessed the effect of VK deficiency on cholestasis in animal models. Akimoto et al. investigated the consequences of common bile duct ligation (BDL) in rats, and attempted to expand the lifespan by feeding a diet supplemented with nutrients [58]. Altered bile secretion due to BDL impairs VK absorption, leading to VK deficiency. This study also demonstrated that significantly lower plasma VK_1_ levels in BDL rats than those in sham-operated rats resulted in massive hemorrhaging in body cavities or organs, which was the direct cause of death [58].

## 7. Vitamin K Supplementation in Cholestasis and Other Hepatic Diseases

VK supplementation is generally believed to be essential to manage the liver disease, as VK helps in preventing bleeding. Moreover, bile is enriched in bile salts, which are necessary for the absorption of VK and other fat-soluble vitamins. VK absorption is very low in severe lipid malabsorption syndromes. Therefore, periodic administration of VK intramuscularly or intravenously is necessary for chronic cholestasis and severe liver failure, respectively [61] (Table 1).

In 1995, Beck et al. reported that a weekly dose of 50 μg of VK_1_ subcutaneously improved the mortality rate of BDL Sprague–Dawley rats from 20–25% to 10% [62]. The authors suggested that this improvement was due to a reduction in hemorrhagic complications, as there was no change in serum biochemical parameters. In 2005, Akimoto et al. showed increased (statistically nonsignificant) lifespans of BDL Sprague–Dawley rats fed with a nutrient-supplemented diet including VK_3_ [58]. The authors identified massive hemorrhage as the main cause of death in animals that developed cirrhosis within four weeks of common BDL. VK may have contributed to the prevention of hemorrhage in rats fed VK_3_ containing a nutritionally enriched diet feeding group [58]. Jiao et al. evaluated the effect of VK_1_ on alleviating BDL-induced fibrosis at the histological and biochemical levels during the 28-day experiment. The results of this study indicated that the severity of lesions can be reduced by VK_1_ treatment. The authors considered the potential role of the VK_1_-mediated activation of PXR to protect mice from cholestasis, because VK can activate PXR, and PXR is reported to protect against cholestasis. However, further studies are needed to show that VK_1_ does not delay the disease process [47]. Furthermore, we previously demonstrated that mRNA levels of *Cyp7a1* and *Cyp8b1,* which encode two key enzymes in BA synthesis, were significantly suppressed by MK-4 treatment in humanized PXR mice, but not in wild-type (WT) mice. Moreover, MK-4 treatment significantly suppressed both *CYP7A1* and *CYP8B1* mRNA levels in HepG2 cells [8].

In neonatal cholestasis, along with other nutritional supplements, oral VK_1_ is recommended at a dose ranging from 2.5 mg biweekly and 5.0 mg/day as soon as VK deficiency is observed [63]. The American Association for the Study of Liver Diseases (AASLD) recommends that subcutaneous VK should be given therapeutically if INR is found to be prolonged and responds to a VK trial [64]. The European Association for the Study of Liver Disease (EASLD) suggests giving VK supplementation prophylactically in severe cholestasis before any invasive procedure, considering the context of bleeding episodes [65]. Moreover, although the mechanism of action of VK is unknown, China’s 2012 Guidelines for the Diagnosis and Treatment of Liver Failure recommend 5–10 mg of VK_1_ to treat patients with liver failure, as they often have VK deficiency [66]. In 2018, a slight modification of the guidelines stated that in cases featuring bleeding, 5–10 mg of VK_1_ can be used for a short time when there is VK deficiency [67]. Furthermore, osteoporosis is commonly diagnosed in patients with PBC because of the malabsorption of fat-soluble vitamins caused by the reduced secretion of BAs. PBC is prevalent in middle-aged and postmenopausal women. Therefore, along with VD_3_, VK_2_ formulations are frequently prescribed for PBC in Japan [50].

In 1992, Amedee-Manesme et al. compared two different types of VK_1_ solution: Konakion formulation and mixed micelles Konakion (MM) formulation. These were prepared with VK_1_ solubilized in glycocholate and lecithin for the treatment of cholestasis in children. The MM solution efficiently and safely corrected VK deficiency [61]. A randomized pilot trial of MK-4 for bone loss in female patients with PBC and with low bone mineral density (BMD) was conducted [68]. BMD increased after one year of MK-4 treatment, but returned to near baseline level after two years. However, BMD was significantly higher in the MK-4 treated group than in the control group throughout the two years of treatment [68]. A small study on cholestatic patients demonstrated that VK therapy (7.8–700 μg/kg/day) was positively correlated with the severity of cholestasis, but no correlation was found with PT, INR, or protein induced by VK absence or antagonist-II (PIVKA-II) levels, suggesting a need for investigating a better strategy for VK supplementation [57]. A recent retrospective cohort study showed that VK_1_ may reduce the risk of death in Chinese patients with chronic liver failure [69]. This study analyzed the effect of intramuscular injection of VK_1_ treatment according to the 2012 Guidelines for the Diagnosis and Treatment of Liver Failure in China in patients with different types of liver disease, including cholestatic liver disease. Analysis of survival at 48 weeks revealed that VK_1_ reduced the INR level as well as mortality in patients with chronic liver failure [69].

It has been reported that 20–40% of patients with cirrhosis have coagulation abnormalities [70], and have an increased risk of bleeding and clotting because of the decreased synthetic capabilities of the cirrhotic liver. Therefore, VK is routinely recommended to correct prolonged PT in patients with cirrhosis. Supplementation of VK in various ways has been reported for the treatment of liver disease in humans since 1988 [71]. It has been reported that the combination treatment using VK_1_ and BA, particularly ursodeoxycholic acid (UDCA), is useful to reduce the hemorrhagic tendency in patients with decompensated liver cirrhosis, whereas VK_1_ alone failed to improve the hemorrhagic tendency [71]. In 2002, Shiomi et al. evaluated the effects of MK-4 treatment in women with osteoporosis associated with liver cirrhosis [72]. The patients had underlying hepatitis viral infections. BMD increased after one year of treatment with 45 mg/day of MK-4 in capsule form, but returned to near the baseline level after two years of treatment. However, BMD continued to be significantly higher in the treated group than in the control group throughout the entire study period [72]. Habu et al. reported that MK-4 may have a protective role in the prevention of hepatocellular carcinoma (HCC) in women with viral cirrhosis [73]. In this study, 45 mg/day of MK-4 was administered to the treatment group to prevent bone loss. In 2004, Otsuka et al. demonstrated that a high dose of MK-4 inhibits the growth and invasiveness of HCC cells by PKA activation [74]. The authors showed that after subcutaneous tumor formation, VK_2_ treatment prevented body weight loss, and the size of the tumors was smaller in MK-4 treated mice than in the control mice. In another study, a combination treatment of MK-4 and the angiotensin-converting enzyme inhibitor perindopril (PE) was an effective strategy for chemoprevention against HCC in rats and humans [75,76]. Several studies have tested the effects of MK-4 on recurrent HCC and survival after curative treatment [77,78,79,80,81,82,83,84]. Some of these studies have shown that MK-4 may have a reducing effect on the recurrence of HCC and a favorable effect on survival [77,78,81,83], although some studies have found no significant effect [79,80,84].

In contrast, some studies demonstrated that VK cannot be used in patients with liver disease [85,86,87,88,89]. A retrospective study of patients with cirrhosis reported that VK was not useful for cirrhosis, but could be supplemented parenterally only during cholestasis [85]. In a placebo-controlled trial of VK supplementation on BMD in PBC, one group of patients was treated with 2 mg/day of VK orally for one year [86]. All patients received oral calcium at 1 g/day and VD at 20 μg/day for one month prior to randomization and continued throughout the study. No significant effect of VK treatment was found in BMD of the spine (L2–L4) or femoral neck [86]. Saja et al. found that VK was not able to significantly improve the majority of coagulation parameters in patients with liver disease [87]. However, no patient with cholestasis was included in the study. Furthermore, this study only administered a single dose of VK_1_. Another retrospective study evaluated the effectiveness of intravenous VK therapy in patients with cirrhosis [88]. The effectiveness of therapy was defined as a 30% decrease in INR or a reduction in INR to an absolute value of ≤1.5. Of the patients, 62.3% failed to achieve at least a 10% decrease, and only 16.7% met the primary effectiveness endpoint. The authors concluded that the use of intravenous VK to correct coagulopathy in cirrhosis may not be beneficial. However, this study evaluated a severely ill cirrhotic population. Therefore, the results may not be generalizable to all patients with cirrhosis [88]. Furthermore, Aldrich et al. demonstrated that the routine use of VK has no beneficial effect in the correction of cirrhosis-related coagulopathy [89]. However, this study did not consider cholestasis in pediatric patients. Therefore, in agreement with Xiong et al., we would suggest that cholestasis could be the cause of inconsistency in some research conclusions [69].

## 8. Potential Role of Vitamin K on Cholestatic Liver Disease

The potential role of VK in ameliorating the complications of cholestatic liver disease in the context of the mode of action of VK is discussed here.

### 8.1. Post-Translational Modifications (Gla Protein Formation)

Interestingly, warfarin, which inhibits VK function, has been in use as an anti-coagulant since 1954, before the revealing of the necessity of VK for γ-carboxylation in some coagulation factors, and in many countries, VK has been used to prevent intracranial hemorrhage in newborn babies since 1960 [2,16]. Buitenhuis et al. showed that MK-3 had the highest cofactor activity, whereas VK_1_ and MK-4 had almost similar cofactor activity in their study conditions [90]. Coagulation factors II, VII, IX, and X, as well as anti-coagulation proteins C, S, and Z, are well-known VKD proteins [91]. VK appears to be essential in liver diseases, because it can contribute to the prevention of bleeding in liver tissues. VK reportedly improves the mortality rate of rats by reducing hemorrhagic complications [58,62].

In 1960, it was reported that VK plays an important role in accelerating the rate of bone healing in rats and rabbits [92]. In 1985, Hart et al. reported that low levels of circulating VK_1_ in plasma were associated with the risk of bone fractures [93]. This association has been further evaluated in several studies [94,95,96]. VKD proteins, such as osteocalcin, matrix Gla protein (MGP), growth arrest-specific protein 6, and Gla-rich protein, play important roles in modulating bone [97,98,99]. It has been reported that a high amount of VK_1_ is required for maximal osteocalcin γ-carboxylation [98]. In 2011, it was reported that MK-4 induces osteoblastogenesis and reduces osteoclastogenesis by suppressing NF-κB activation and increasing IκB mRNA in a γ-carboxylation-independent manner [100]. NF-κB signaling has two functions in bone metabolism: it stimulates osteoclast development and resorption while inhibiting osteoblast differentiation and activity.

In osteoporosis, bone density is decreased, eventually resulting in an increased risk of fractures [101]. Based on domestic clinical trials, Japan approved MK-4 as a drug for osteoporosis in 1995 [102]. Later, many interventional clinical trials have been conducted worldwide using VK_1_, MK-4, or MK-7 [97]. Although most of these clinical trials have been conducted in postmenopausal women, experimental evidence indicates the necessity of VK to prevent osteoporosis.

Osteoporosis is a common complication in different forms of liver disease. It is four times more prevalent in patients with PBC than in controls [103]. Morbidity and mortality in patients with chronic liver diseases, including PBC, can be increased if osteoporosis is not treated in time. The AASLD and EASLD suggest calcium and VD supplementation in patients with PBC to prevent osteoporosis [64,65]. Current treatment options for PBC are mostly derived from postmenopausal patients without PBC. Probably because of the difference in the pathophysiological mechanisms of these two diseases, the therapies have been found to be less effective in PBC. Postmenopausal osteoporosis is primarily due to increased bone resorption, whereas osteoporosis in PBC is mostly due to reduced bone formation. A recent systematic review and meta-analysis of treatments for osteoporosis demonstrated that none of the studies met the primary outcome of fracture reduction or improvement in BMD. Therefore, new interventions for improving bone formation in patients with PBC are essential [101].

### 8.2. Pregnane X Receptor Activation

**Figure 2:**
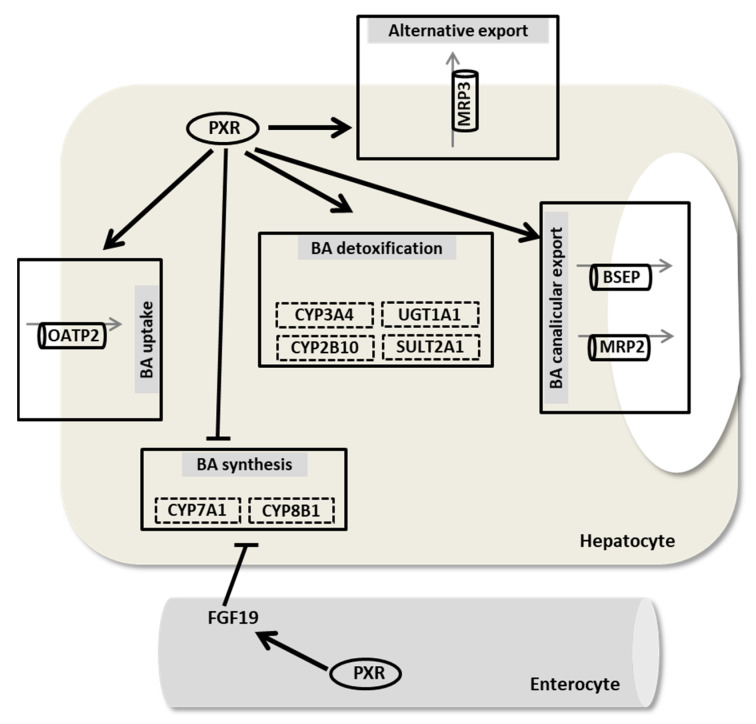
The role of PXR in bile acid metabolism in humans. PXR represses BA synthesis by inhibiting the expression of CYP7A1 and CYP8B1. Intestinal PXR activates FGF19, which in turn inhibits BA synthesis. PXR induces BA detoxification (CYP3A4, CYP2B10, and SULT2A1) and stimulates bilirubin conjugation (UGT1A1) and excretion (MRP2, BSEP, and MRP3). Additionally, PXR can induce BA uptake via OATP2. PXR, pregnane X receptor; OATP, organic anion transporting polypeptide; BSEP, bile salt export pump; MRP, multidrug resistance protein; FGF, fibroblast growth factor; CYP7A1, cholesterol 7 α-hydroxylase; CYP8B1, sterol 12-α-hydroxylase; CYP3A4, cytochrome P450 3A4, CYP2B10, cytochrome P450 2B10; UGT1A1, uridine diphosphate glucuronosyltransferase; and SULT2A1, sulfotransferase 2A1. Bold black arrows indicate induction; bars indicate inhibition; ellipses denote receptors; cylinders denote transporters; and broken line boxes denote enzymes.

It has been reported that after BDL-induced cholestasis, PXR-deficient mice exhibited more hepatic damage (large areas of hepatic necrosis and bile infarcts) than WT mice [104]. Another study demonstrated that the activation of PXR by its ligand reduced bilirubin and serum levels of BAs by inducing phase I, II, and III detoxification systems [105]. Furthermore, PXR is a transcriptional target of farnesoid X receptor (FXR), which is the key regulator of BA synthesis [25]. Common genetic polymorphisms of PXR are also associated with increased susceptibility to intrahepatic cholestasis of pregnancy and PSC [106,107]. Functional PXR gene polymorphisms influence disease progression in patients with PSC [107]. Chen et al. demonstrated that the expression of transporters and NRs differ in the early and late stages of cholestasis. Increased expression of PXR and constitutive androstane receptor (CAR), another NR responsible for xenobiotic metabolism, was observed in patients with obstructive cholestasis, and significant downregulation of PXR was observed in late-stage cholestasis, limiting the progression of liver injury [108]. PXR activation may modulate BA metabolism to rectify cholestasis. Moreover, the inhibition of osteoporosis and inflammation may have an additive effect in ameliorating cholestatic liver diseases. The involvement of PXR in the regulation of transporters and enzymes involved in BA metabolism is shown in Figure 2.

The function of PXR in BA homeostasis was first reported in 2001, when it was suggested that LCA and its metabolite, 3-keto-LCA, can directly activate both mouse and human PXR [30,109]. These studies showed that the administration of LCA, a highly toxic secondary BA formed in the intestine, may cause intrahepatic cholestasis. Pharmacological stimulation of PXR improves LCA-induced liver toxicity. When activated by LCA and its metabolite, PXR inhibits *Cyp7a1* that blocks BA synthesis and increases the uptake of LCA and other BAs from sinusoidal blood into the hepatocytes, leading to hydroxylation by Cyp3a enzymes facilitating excretion [55]. Therefore, PXR activation by LCA seems to be adaptive endogenous protection to reduce BA toxicity in cholestasis [110]. Another study reported that the activation of PXR by PCN strongly induced the BA-hydroxylating enzymes Cyp3a11 (in human CYP3A4) and Cyp2b10 [105]. It was demonstrated that PXR activation regulates the biosynthesis, transport, and metabolism of BAs in mice by modulating several genes involved in these processes [30]. Hepatic nuclear factor 4α (HNF4α) and its coactivator, peroxisome proliferator-activated receptor γ coactivator α (PGC1α), are important transcription factors for the transcription of *CYP7A1* and *CYP8B1*. Bhalla et al. suggested that ligand-activated PXR interacts with PGC1α, stimulating its dissociation from HNF4α on the promoters of *CYP7A1* and *CYP8B1* in HepG2 cells [111]. However, another report demonstrated that ligand-activated PXR interacts with HNF4α, triggering the release of PGC1α to inhibit the transcription of *CYP7A1* in human primary hepatocytes [112]. In the intestine, the activation of PXR induces fibroblast growth factor 15 (*Fgf15*; *FGF19* in humans), which inhibits BA synthesis by reducing the transcription of *Cyp7a1* in the liver [110]. In 2009, it was demonstrated that *CYP3A4* promoter activity was enhanced by MK-4 mediated stimulation of PXR. In 2018, we showed that MK-4 treatment significantly inhibited *Cyp7a1* mRNA expression in humanized PXR mice, but not in WT mice. Furthermore, we reported that *CYP7A1* mRNA expression was suppressed by treatment with MK-4 in HepG2 cells [8].

Moreover, PXR is a regulator of uridine diphosphate glucuronosyltransferase (UGT1A1), an important phase II enzyme for bilirubin glucuronidation and sulfotransferase 2A1 (SUL2A1), and hydroxysteroid sulfotransferase, which increases the solubility of BAs [105,113]. In both PSC and PBC, increased PXR protein was observed compared to the controls, followed by a significant increase of SULT2A1 only in PBC, but not in PSC [114].

Staudinger et al. reported that PCN treatment significantly induced Na-independent organic anion transporter 2 (*Oatp2*) expression in WT mice, but not in PXR knockout mice [30]. *Oatp2* is a basolateral transporter involved in the hepatocellular uptake of a broad-spectrum of amphipathic substrates, including BAs. The canalicular multi-specific organic anion transporter (cMOAT, multidrug resistance protein 2, or MRP2) can transport various compounds, including bilirubin diglucuronide, sulfates, some BAs (e.g., conjugates of LCA), xenobiotics, and their glutathione conjugates into bile; therefore, it is a major determinant of BA-independent bile flow [115]. A significant role of PXR in the regulation of *MRP2* in animals and humans have been reported in different studies [116,117,118]. Treatment with Rif resulted in a strong induction of *Mrp2* mRNA in the livers of male and female rhesus monkeys [117]. Another study reported that dexamethasone, another ligand of PXR, was found to induce *Mrp2* mRNA levels in rat primary hepatocytes [118]. Furthermore, Rif has been reported to play an important role in the induction of *MRP2* mRNA and protein levels in the human small intestine [119]. Teng et al. found induction of *Mrp2* mRNA and protein levels in the liver of WT mice, but not in *Pxr*-deficient mice after the administration of PCN [116]. Moreover, PCN ameliorated hepatic damage in WT mice by inducing *Cyp3a11* and *Mrp3*, but not in *Pxr* knockout mice [30,120]. *Mrp3* may protect the liver from cholestatic injury by reducing the BA concentration in the liver and preventing apoptosis or necrosis [120]. Furthermore, Pxr plays a role in the mechanism of downregulation of *Mrp2*, *Bsep*, and *Cyp3a11* during inflammation in mice [116]. Moreover, it has recently been reported that the activation of PXR and CAR downregulates BA-metabolizing bacteria in the intestine, thereby modulating BA homeostasis [121].

PXR has anti-cholestatic, anti-fibrotic, and anti-inflammatory effects. Pxr activation reduced the levels of inflammatory cytokines, such as tumor necrosis factor alpha (TNFα), in the liver of SJL/J mice. These mice have constitutively high levels of hepatic portal tract inflammatory cell recruitment [122]. This study has also demonstrated that activated Pxr inhibited NF-κB activation, and thus displayed an anti-inflammatory effect. In association with this, another study demonstrated that the anti-inflammatory effect of PXR could be mediated by enhancing the transcription levels of IkBα, thereby inhibiting NF-κB activity [123]. Other authors described that Pxr activation by PCN was able to inhibit carbon tetrachloride-induced fibrosis in mice [124]. Furthermore, *Pxr* knockout mice showed impaired hepatic proliferation, indicating the importance of Pxr in liver regeneration [125].

In 2003, it was reported that MK-4 exerts its effect on the induction of bone markers by γ-carboxylation and transcriptional activation of PXR [3]. The study demonstrated that MK-4 induced the expression of the osteoblastic marker genes MGP and osteoprotegerin by the activation of PXR. It has been demonstrated that MK-4 plays an important role in bone remodeling by transcriptionally regulating tsukushi (*TSK*), matrilin-2 (*MATN2*), and *CD14* in osteoblastic MG63 cells in a PXR-dependent pathway. *TSK* encodes a protein that enhances the accumulation of collagen; MATN2 is a protein comprising extracellular matrix proteins, such as collagen; and CD14 regulates osteoblastogenesis and osteoclastogenesis by inducing differentiation of B cells [41,97].

### 8.3. Anti-Inflammatory Effects

Pathophysiological levels of BAs induce the production of proinflammatory mediators in hepatocytes that initiate inflammation and trigger cholestatic liver injury [53]. However, uncontrolled inflammatory processes can induce further liver injury by damaging the local tissue through the release of soluble mediators and deleterious factors. Detrimental inflammation can be considered both a cause and consequence of cholestasis [126]. The cholestatic liver injury involves several inflammatory pathways, such as the NF-κB, signal transducer, and activator of transcription 3, as well as c-Jun N-terminal kinase pathways [127]. In vivo, the NF-κB transcription factor is a potential master regulator of hepatic inflammation, fibrosis, and the development of HCC [128]. In 2001, it was reported that NF-κB is activated in hepatocytes during obstructive cholestasis, and functions to reduce liver injury in BDL mice. The inhibition of NF-κB potentiated cholestasis-associated liver injury [129]. Activated NF-κB potentiates the production and secretion of proinflammatory cytokines, such as TNF-α and interleukin-6, which are considered to be the promoters of fibrosis and HCC [128,130]. Moreover, it was recently reported that the activation of hepatocyte NF-κB in parenteral nutrition-associated cholestasis may interfere with FXR and liver X receptor signaling, leading to the transcriptional suppression of bile and sterol transporters, such as MRP2, resulting in cholestasis [131]. Therefore, although NF-κB activation is necessary to protect the liver from injury, persistent activation is associated with an increased risk of hepatic fibrosis and HCC [128]. A series of studies have shown the ability of NF-κB inhibitors to stimulate the resolution of fibrosis and regeneration of normal liver tissue in rats [132,133,134]. In 2007, it was demonstrated that MK-4 inhibits the growth of HCC cells by reducing cyclin D1 expression through the IKK/IκB/NF-κB pathway [135,136]. We also demonstrated that the anti-inflammatory activity of VK is mediated by the inactivation of the NF-κB signaling pathway in mouse and human macrophage cells [4,20].

## 9. Conclusions

**Figure 3:**
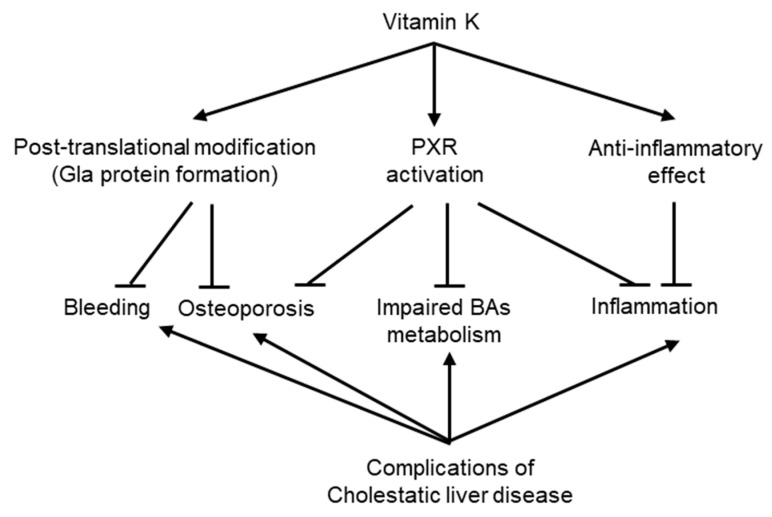
Potential roles of vitamin K in cholestatic liver disease. VK plays several important roles to ameliorate the complications of cholestatic liver disease, at least through three modes of action—posttranslational modification, which allows the formation of several important Gla proteins, leading to the suppression of bleeding and osteoporosis; PXR activation, which may reduce osteoporosis and inflammation, as well as correct BA metabolism; and an anti-inflammatory effect.

The results of clinical trials are not conclusive. Because of the absence of clinical evidence, there are no conclusive guidelines on the use of VK in liver failure. The efficacy of VK in cholestatic liver disease needs to be investigated in large clinical trials with sufficient statistical strength to detect true and clinically meaningful effects. At the same time, several points of experimental evidence indicate that VK plays an important role in reducing the severity of cholestatic liver disease and the risk of mortality, as we have summarized in Figure 3, and that there is no harm reported in the VK treatment; therefore, VK treatment would be suggested for liver failure, particularly in cholestatic liver disease.

## Figures and Tables

**Table 1:** Supplementation of vitamin K in cholestatic liver disease.

Subject	Dose-Duration	Outcome	Ref.	Year
Animal Studies				
Males and females BDL Sprague–Dawley rats	First dose = 50 µg of VK_1_, subcutaneously at the time of operation, and the same dose once per week thereafter for two years	In four weeks, the mortality rate decreased from approximately 20–25% to 10%. There was no difference in the extent of hepatic damage or any hemodynamic or biochemical parameters between VK-treated and untreated rats. The reduction in mortality rate was possibly due to a reduction in hemorrhagic complications, contributing to excess mortality.	[62]	1995
Male BDL Sprague–Dawley rats	MF or NMF diet supplemented with VK_3_ and VD Survival experiment was done until 50 days.	Supplementary VK in the diet ameliorated massive internal hemorrhage and prolonged the survival period.	[58]	2005
Male BDL Sprague–Dawley rats	After BDL, one group of rats was treated by intramuscular injection of VK_1_ once per week at a dose of 8 mg/kg for four weeks. Drinking water containing gentamicin (160 mg/L) was given to all animals.	The levels of biochemical parameters, fibrotic score, collagen content, α-SMA, and CK19 expression were significantly reduced by treatment with VK_1_.	[47]	2014
**Human Studies**				
1–6 months infant with cholestasis	Single dose of 10 mg of VK_1_ or 10 mg of Konakion biweekly for six months, followed by 10 mg of MM solution, a formulation of VK solubilized in glycocholate and lecithin, biweekly either orally or intramuscularly for over three months	Konakion (VK_1_) MM efficiently and safely corrected VK deficiency	[61]	1992
Human	Not known	VK was not useful for cirrhosis, but can be supplemented parenterally only during cholestasis	[85]	1999
Women with PBC	All were administered UDCA (600 mg/day) during hospitalization. Half of the patients were randomly selected to receive 45 mg/day of MK-4 orally for at least two years.	BMD increased after one year of treatment with MK-4, but returned to near the baseline after two years. However, BMD continued to be significantly higher in the treated group than in the control group throughout the two years of treatment.	[68]	2001
Patients with PBC	2 mg/day of VK orally for 12 months. All the patients received oral calcium (1 g/day) and VD (20 μg/day) for one month prior to randomization and continued throughout the study. BMD scanning of the spine (L2–L4) and femoral neck was performed at 0 and 12 months.	No significant effect of VK treatment was found.	[86]	2003
Patients with cholestasis	7.8–700 μg/kg/day of oral VK The duration of the supplementation is not known.	VK intake was positively correlated with the severity of cholestasis. No correlation was found with PT, INR, and PIVKA-II levels.	[57]	2009
Patients with chronic liver failure	Daily intramuscular injection of 10 mg of VK_1_ followed up for 48 weeks	VK_1_ reduced the INR levels as well as the risk of death	[69]	2020

BDL, bile duct ligation; VK, vitamin K; MK-4, menaquinone-4; VD, vitamin D; α-SMA, α-smooth muscle actin; CK19, cytokeratin 19; UDCA, ursodeoxycholic acid; BMD, bone mineral density; PT, prothrombin time; INR, international normalized ratio; PIVKA-II, protein induced by vitamin K absence or antagonist-II.

## Data Availability

Not applicable.

## References

[1] Stafford D.W. (2005). The vitamin K cycle. J. Thromb. Haemost..

[2] Azuma K., Inoue S. (2017). Vitamin K, SXR, and GGCX. Vitamin K2-Vital for Health and Wellbeing.

[3] Tabb M.M., Sun A., Zhou C., Grun F., Errandi J., Romero K., Pham H., Inoue S., Mallick S., Lin M. (2003). Vitamin K2 Regulation of Bone Homeostasis Is Mediated by the Steroid and Xenobiotic Receptor SXR. J. Biol. Chem..

[4] Ohsaki Y., Shirakawa H., Miura A., Giriwono P.E., Sato S., Ohashi A., Iribe M., Goto T., Komai M. (2010). Vitamin K suppresses the lipopolysaccharide-induced expression of inflammatory cytokines in cultured macrophage-like cells via the inhibition of the activation of nuclear factor κB through the repression of IKKα/β phosphorylation. J. Nutr. Biochem..

[5] Ito A., Shirakawa H., Takumi N., Minegishi Y., Ohashi A., Howlader Z.H., Ohsaki Y., Sato T., Goto T., Komai M. (2011). Menaquinone-4 enhances testosterone production in rats and testis-derived tumor cells. Lipids Health Dis..

[6] Lamson D.W., Plaza S.M. (2003). The Anticancer Effects of Vitamin K. Altern. Med. Rev..

[7] Farhadi M.B., Fereidoni M. (2020). Neuroprotective effect of menaquinone-4 (MK-4) on transient global cerebral ischemia/reperfusion injury in rat. PLoS ONE.

[8] Sultana H., Watanabe K., Rana M.M., Takashima R., Ohashi A., Komai M., Shirakawa H. (2018). Effects of Vitamin K_2_ on the Expression of Genes Involved in Bile Acid Synthesis and Glucose Homeostasis in Mice with Humanized PXR. Nutrients.

[9] Beulens J.W., van der A D.L., Grobbee D.E., Sluijs I., Spijkerman A.M., van der Schouw Y.T. (2010). Dietary Phylloquinone and Menaquinones Intakes and Risk of Type 2 Diabetes. Diabetes Care.

[10] Neogi T., Booth S.L., Zhang Y.Q., Jacques P.F., Terkeltaub R., Aliabadi P., Felson D.T. (2006). Low Vitamin K Status Is Associated with Osteoarthritis in the Hand and Knee. Arthritis Rheum..

[11] Presse N., Shatenstein B., Kergoat M.J., Ferland G. (2008). Low Vitamin K Intakes in Community-Dwelling Elders at an Early Stage of Alzheimer’s Disease. J. Am. Diet Assoc..

[12] Geleijnse J.M., Vermeer C., Grobbee D.E., Schurgers L.J., Knapen M.H., van der Meer I.M., Hofman A., Witteman J.C. (2004). Dietary Intake of Menaquinone Is Associated with a Reduced Risk of Coronary Heart Disease: The Rotterdam Study. J. Nutr..

[13] Yasin M., Butt M.S., Zeb A. (2017). Vitamin K2 Rich Food Products. Vitamin K2-Vital for Health and Wellbeing.

[14] Thijssen H.H., Drittij-Reijnders M.J. (1996). Vitamin K status in human tissues: Tissue-specific accumulation of phylloquinone and menaquinone-4. Br. J. Nutr..

[15] Yamamoto R., Komai M., Kojima K., Furukawa Y., Kimura S. (1997). Menaquinone-4 accumulation in various tissues after an oral administration of phylloquinone in Wistar rats. J. Nutr. Sci. Vitaminol. (Tokyo).

[16] Nelsestuen G.L., Zytkovicz T.H., Howard J.B. (1974). The mode of action of vitamin K. Identification of gamma-carboxyglutamic acid as a component of prothrombin. J. Biol. Chem..

[17] Stenflo J., Fernlund P., Egan W., Roepstor P. (1974). Vitamin K dependent modifications of glutamic acid residues in prothrombin. Proc. Natl. Acad. Sci. USA.

[18] Blumberg B., Sabbagh W., Juguilon H., Bolado J., van Meter C.M., Ong E.S., Evans R.M. (1998). SXR, a novel steroid and xenobiotic-sensing nuclear receptor. Genes Dev..

[19] Ohsaki Y., Shirakawa H., Hiwatashi K., Furukawa Y., Mizutani T., Komai M. (2006). Vitamin K suppresses lipopolysaccharide-induced inflammation in the rat. Biosci. Biotechnol. Biochem..

[20] Saputra W.D., Aoyama N., Komai M., Shirakawa H. (2019). Menaquinone-4 Suppresses Lipopolysaccharide-Induced Inflammation in MG6 Mouse Microglia-Derived Cells by Inhibiting the NF-κB Signaling Pathway. Int. J. Mol. Sci..

[21] Azuma K., Inoue S. (2019). Multiple Modes of Vitamin K Actions in Aging-Related Musculoskeletal Disorders. Int. J. Mol. Sci..

[22] Kliewer S.A., Moore J.T., Wade L., Staudinger J.L., Watson M.A., Jones S.A., McKee D.D., Oliver B.B., Willson T.M., Zetterstrom R.H. (1998). An Orphan Nuclear Receptor Activated by Pregnanes Defines a Novel Steroid Signaling Pathway. Cell.

[23] Bertilsson G., Heidrich J., Svensson K., Asman M., Jendeberg L., Sydow-Backman M., Ohlsson R., Postlind H., Blomquist P., Berkenstam A. (1998). Identification of a human nuclear receptor defines a new signaling pathway for CYP3A induction. Proc. Natl. Acad. Sci. USA.

[24] Pavek P. (2016). Pregnane X Receptor (PXR)-Mediated Gene Repression and Cross-Talk of PXR with Other Nuclear Receptors via Coactivator Interactions. Front. Pharmacol..

[25] Jung D., Mangelsdorf D.J., Meyer U.A. (2006). Pregnane X receptor is a target of farnesoid X receptor. J. Biol. Chem..

[26] Watkins R.E., Davis-Searles P.R., Lambert M.H., Redinbo M.R. (2003). Coactivator Binding Promotes the Specific Interaction Between Ligand and the Pregnane X Receptor. J. Mol. Biol..

[27] Watkins R.E., Wisely G.B., Moore L.B., Collins J.L., Lambert M.H., Williams S.P., Willson T.M., Kliewer S.A., Redinbo M.R. (2001). The Human Nuclear Xenobiotic Receptor PXR: Structural Determinants of Directed Promiscuity. Science.

[28] Watkins R.E., Maglich J.M., Moore L.B., Wisely G.B., Noble S.M., Davis-Searles P.R., Lambert M.H., Kliewer S.A., Redinbo M.R. (2003). 2.1 Å Crystal Structure of Human PXR in Complex with the St. John’s Wort Compound Hyperforin. Biochemistry.

[29] Zhou C., Verma S., Blumberg B. (2009). The steroid and xenobiotic receptor (SXR), beyond xenobiotic metabolism. Nucl. Recept. Signal..

[30] Staudinger J.L., Goodwin B., Jones S.A., Hawkins-Brown D., MacKenzie K.I., LaTour A., Liu Y., Klaassen C.D., Brown K.K., Reinhard J. (2001). The nuclear receptor PXR is a lithocholic acid sensor that protects against liver toxicity. Proc. Natl. Acad. Sci. USA.

[31] Guengerich F.P. (1999). Cytochrome P-450 3A4: Regulation and role in drug metabolism. Ann. Rev. Pharmacol. Toxicol..

[32] Kliewer S.A., Willson T.M. (2002). Regulation of xenobiotic and bile acid metabolism by the nuclear pregnane X receptor. J. Lipid Res..

[33] Chrencik J.E., Orans J., Moore L.B., Xue Y., Peng L., Collins J.L., Wisely G.B., Lambert M.H., Kliewer S.A., Redinbo M.R. (2005). Structural disorder in the complex of human pregnane X receptor and the macrolide antibiotic rifampicin. Mol. Endocrinol..

[34] Carnahan V.E., Redinbo M.R. (2005). Structure and function of the human nuclear xenobiotic receptor PXR. Curr. Drug Metab..

[35] Banerjee M., Chen T. (2013). Differential regulation of CYP3A4 promoter activity by a new class of natural product derivatives binding to pregnane X receptor. Biochem. Pharmacol..

[36] Jones S.A., Moore L.B., Shenk J.L., Wisely G.B., Hamilton G.A., McKee D.D., Tomkinson N.C., LeCluyse E.L., Lambert M.H., Willson T.M. (2000). The Pregnane X Receptor: A Promiscuous Xenobiotic Receptor That Has Diverged during Evolution. Mol. Endocrinol..

[37] Xie W., Barwick J.L., Downes M., Blumberg B., Simon C.M., Nelson M.C., Neuschwander-Tetri B.A., Brunt E.M., Guzelian P.S., Evans R.M. (2000). Humanized xenobiotic response in mice expressing nuclear receptor SXR. Nature.

[38] Ma X., Shah Y., Cheung C., Guo G.L., Feigenbaum L., Krausz K.W., Idle J.R., Gonzalez F.J. (2007). The Pregnane X Receptor Gene-Humanized mouse: A Model for Investigating drug-drug Interactions Mediated by Cytochromes P450 3A. Drug Metab. Dispos..

[39] Scheer N., Ross J., Rode A., Zevnik B., Niehaves S., Faust N., Wolf C.R. (2008). A novel panel of mouse models to evaluate the role of human pregnane X receptor and constitutive androstane receptor in drug response. J. Clin. Investig..

[40] Igarashi K., Kitajima S., Aisaki K., Tanemura K., Taquahashi Y., Moriyama N., Ikeno E., Matsuda N., Saga Y., Blumberg B. (2012). Development of humanized steroid and xenobiotic receptor mouse by homologous knock-in of the human steroid and xenobiotic receptor ligand binding domain sequence. J. Toxicol. Sci..

[41] Ichikawa T., Horie-Inoue K., Ikeda K., Blumberg B., Inoue S. (2006). Steroid and Xenobiotic Receptor SXR Mediates Vitamin K2-activated Transcription of Extracellular Matrix-related Genes and Collagen Accumulation in Osteoblastic Cells. J. Biol. Chem..

[42] Azuma K., Urano T., Ouchi Y., Inoue S. (2009). Vitamin K2 Suppresses Proliferation and Motility of Hepatocellular Carcinoma Cells by Activating Steroid and Xenobiotic Receptor. Endocr. J..

[43] Avior Y., Levy G., Zimerman M., Kitsberg D., Schwartz R., Sadeh R., Moussaieff A., Cohen M., Itskovitz-Eldor J., Nahmias Y. (2015). Microbial-derived lithocholic acid and vitamin K2 drive the metabolic maturation of pluripotent stem cells-derived and fetal hepatocytes. Hepatology.

[44] Chiang J.Y. (2009). Bile acids: Regulation of synthesis. J. Lipid Res..

[45] Trauner M., Meier P.J., Boyer J.L. (1998). Molecular pathogenesis of cholestasis. N. Engl. J. Med..

[46] Samant H., Manatsathit W., Dies D., Shokouh-Amiri H., Zibari G., Boktor M., Alexander J.S. (2019). Cholestatic liver diseases: An era of emerging therapies. World J. Clin. Cases.

[47] Jiao K., Sun Q., Chen B., Li S., Lu J. (2014). Vitamin K1 attenuates bile duct ligation-induced liver fibrosis in rats. Scand. J. Gastroenterol..

[48] European Association for the Study of the Liver (2009). EASL Clinical Practice Guidelines: Management of cholestatic liver diseases. J. Hepatol..

[49] Appanna G., Kallis Y. (2020). An update on the management of cholestatic liver diseases. Clin. Med. (Lond.).

[50] Working Subgroup (English version) for Clinical Practice Guidelines for Primary Biliary Cirrhosis (2014). Guidelines for the management of primary biliary cirrhosis: The Intractable Hepatobiliary Disease Study Group supported by the Ministry of Health, Labour and Welfare of Japan. Hepatol. Res..

[51] Alvelius G., Hjalmarson O., Griffiths W.J., Bjorkhem I., Sjovall J. (2001). Identification of unusual 7-oxygenated bile acid sulfates in a patient with Niemann-Pick disease, type C. J. Lipid Res..

[52] Hofmann A.F. (2002). Cholestatic liver disease: Pathophysiology and therapeutic options. Liver.

[53] Cai S.Y., Li M., Boyer J.L., Arias I.M., Alter H.J., Boyer J.L., Cohen D.E., Shafritz D.A., Thorgeirsson S.S., Wolkoff A.W. (2020). The Role of Bile Acid-Mediated Inflammation in Cholestatic Liver Injury. The Liver: Biology and Pathobiology.

[54] Higuchi H., Miyoshi H., Bronk S.F., Zhang H., Dean N., Gores G.J. (2001). Bid antisense attenuates bile acid-induced apoptosis and cholestatic liver injury. J. Pharmacol. Exp. Ther..

[55] Jonker J.W., Liddle C., Downes M. (2012). FXR and PXR: Potential therapeutic targets in cholestasis. J. Steroid Biochem. Mol. Biol..

[56] Maldonado M., Alhousseini A., Awadalla M., Idler J., Welch R., Puder K., Patwardhan M., Gonik B. (2017). Intrahepatic Cholestasis of Pregnancy Leading to Severe Vitamin K Deficiency and Coagulopathy. Case Rep. Obstet. Gynecol..

[57] Strople J., Lovell G., Heubi J. (2009). Prevalence of subclinical vitamin K deficiency in cholestatic liver disease. J. Pediatr. Gastroenterol. Nutr..

[58] Akimoto T., Hayashi N., Adachi M., Kobayashi N., Zhang X.J., Ohsuga M., Katsuta Y. (2005). Viability and plasma vitamin K levels in the common bile duct-ligated rats. Exp. Anim..

[59] Kowdley K.V., Emond M.J., Sadowski J.A., Kaplan M.M. (1997). Plasma vitamin K1 level is decreased in primary biliary cirrhosis. Am. J. Gastroenterol..

[60] Mager D.R., McGee P.L., Furuya K.N., Roberts E.A. (2006). Prevalence of vitamin K deficiency in children with mild to moderate chronic liver disease. J. Pediatr. Gastroenterol. Nutr..

[61] Amédée-Manesme O., Lambert W.E., Alagille D., De Leenheer A.P. (1992). Pharmacokinetics and safety of a new solution of vitamin K1(20) in children with cholestasis. J. Pediatr. Gastroenterol. Nutr..

[62] Beck P.L., Lee S.S. (1995). Vitamin K1 improves survival in bile-duct-ligated rats with cirrhosis. J. Hepatol..

[63] Best C., Gourley G.R. (2009). Management of neonatal cholestasis. Therapy.

[64] Lindor K.D., Bowlus C.L., Boyer J., Levy C., Mayo M. (2019). Primary biliary cholangitis: 2018 practice guidance from the American Association for the study of liver diseases. Hepatology.

[65] European Association for the Study of the Liver (2017). EASL Clinical practice guidelines: The diagnosis and management of patients with primary biliary cholangitis. J. Hepatol..

[66] Liver Failure and Artificial Liver Group, Chinese Society of Infectious Diseases, Chinese Medical Association, Severe Liver Diseases and Artificial Liver Group, Chinese Society of Hepatology, Chinese Medical Association (2013). Diagnostic and treatment guidelines for liver failure (2012 version). Zhonghua Gan Zang Bing Za Zhi..

[67] Xiong Z., Liu Y., Chang T., Xu X., Huo S., Deng H., Liu T., Leng Y. (2020). Effect of vitamin K1 on survival of patients with chronic liver failure: A retrospective cohort study. Medicine (Baltimore).

[68] Liver Failure and Artificial Liver Group, Chinese Society of Infectious Diseases, Chinese Medical Association, Severe Liver Disease and Artificial Liver Group, Chinese Society of Hepatology, Chinese Medical Association (2019). Guideline for diagnosis and treatment of liver failure. Zhonghua Gan Zang Bing Za Zhi.

[69] Nishiguchi S., Shimoi S., Kurooka H., Tamori A., Habu D., Takeda T., Kubo S. (2001). Randomized pilot trial of vitamin K2 for bone loss in patients with primary biliary cirrhosis. J. Hepatol..

[70] Shah N.L., Northup P.G., Caldwell S.H. (2012). A clinical survey of bleeding, thrombosis and blood product use in decompensated cirrhosis patients. Ann. Hepatol..

[71] Nambu M., Iijima T. (1988). A combination therapy of vitamin K1 and bile acid on hemorrhagic diathesis in patients with decompensated liver cirrhosis. Gastroenterol. Jpn..

[72] Shiomi S., Nishiguchi S., Kubo S., Tamori A., Habu D., Takeda T., Ochi H. (2002). Vitamin K2 (menatetrenone) for bone loss in patients with cirrhosis of the liver. Am. J. Gastroenterol..

[73] Habu D., Shiomi S., Tamori A., Takeda T., Tanaka T., Kubo S., Nishiguchi S. (2004). Role of vitamin K2 in the development of hepatocellular carcinoma in women with viral cirrhosis of the liver. JAMA.

[74] Otsuka M., Kato N., Shao R.X., Hoshida Y., Ijichi H., Koike Y., Taniguchi H., Moriyama M., Shiratori Y., Kawabe T. (2004). Vitamin K2 inhibits the growth and invasiveness of hepatocellular carcinoma cells via protein kinase A activation. Hepatology.

[75] Yoshiji H., Kuriyama S., Noguchi R., Yoshii J., Ikenaka Y., Yanase K., Namisaki T., Kitade M., Yamazaki M., Masaki T. (2005). Combination of vitamin K2 and the angiotensin-converting enzyme inhibitor, perindopril, attenuates the liver enzyme-altered preneoplastic lesions in rats via angiogenesis suppression. J. Hepatol..

[76] Yoshiji H., Noguchi R., Yamazaki M., Ikenaka Y., Sawai M., Ishikawa M., Kawaratani H., Mashitani T., Kitade M., Kaji K. (2007). Combined treatment of vitamin K2 and angiotensin-converting enzyme inhibitor ameliorates hepatic dysplastic nodule in a patient with liver cirrhosis. World J. Gastroenterol..

[77] Mizuta T., Ozaki I., Eguchi Y., Yasutake T., Kawazoe S., Fujimoto K., Yamamoto K. (2006). The effect of menatetrenone, a vitamin K2 analog, on disease recurrence and survival in patients with hepatocellular carcinoma after curative treatment: A pilot study. Cancer.

[78] Kakizaki S., Sohara N., Sato K., Suzuki H., Yanagisawa M., Nakajima H., Takagi H., Naganuma A., Otsuka T., Takahashi H. (2007). Preventive effects of vitamin K on recurrent disease in patients with hepatocellular carcinoma arising from hepatitis C viral infection. J. Gastroenterol. Hepatol..

[79] Hotta N., Ayada M., Sato K., Ishikawa T., Okumura A., Matsumoto E., Ohashi T., Kakumu S. (2007). Effect of vitamin K2 on the recurrence in patients with hepatocellular carcinoma. Hepatogastroenterology.

[80] Hosho K., Okano J., Koda M., Murawaki Y. (2008). Vitamin K2 Has No Preventive Effect on Recurrence of Hepatocellular Carcinoma after Effective Treatment. Yonago Acta Med..

[81] Yoshiji H., Noguchi R., Toyohara M., Ikenaka Y., Kitade M., Kaji K., Yamazaki M., Yamao J., Mitoro A., Sawai M. (2009). Combination of vitamin K2 and angiotensin-converting enzyme inhibitor ameliorates cumulative recurrence of hepatocellular carcinoma. J. Hepatol..

[82] Yoshida H., Shiratori Y., Kudo M., Shiina S., Mizuta T., Kojiro M., Yamamoto K., Koike Y., Saito K., Koyanagi N. (2011). Effect of vitamin K2 on the recurrence of hepatocellular carcinoma. Hepatology.

[83] Ishizuka M., Kubota K., Shimoda M., Kita J., Kato M., Park K.H., Shiraki T. (2012). Effect of menatetrenone, a vitamin K2 analog, on recurrence of hepatocellular carcinoma after surgical resection: A prospective randomized controlled trial. Anticancer Res..

[84] Riaz I.B., Riaz H., Riaz T., Rahman S., Amir M., Badshah M.B., Kazi A.N. (2012). Role of vitamin K2 in preventing the recurrence of hepatocellular carcinoma after curative treatment: A meta-analysis of randomized controlled trials. BMC Gastroenterol..

[85] Otero Fernandez M.A., Romero-Gomez M., Martinez Delgado C., González Suárez M. (1999). Usefulness of vitamin K in hepatic cirrhosis. Aten. Primaria.

[86] Gleeson D., Hodges S., Rigney E., Blumsohn A., Hannon R., Peel N., Eastell R. (2003). 740 placebo-controlled trial of vitamin K supplementation on bone mineral density in primary biliary cirrhosis (PBC). Hepatology.

[87] Saja M.F., Abdo A.A., Sanai F.M., Shaikh S.A., Gader A.G. (2013). The coagulopathy of liver disease: Does vitamin K help?. Blood Coagul. Fibrinolysis.

[88] Rivosecchi R.M., Kane-Gill S.L., Garavaglia J., MacLasco A., Johnson H. (2017). The effectiveness of intravenous vitamin K in correcting cirrhosis-associated coagulopathy. Int. J. Pharm. Pract..

[89] Aldrich S.M., Regal R.E. (2019). Routine Use of Vitamin K in the Treatment of Cirrhosis-Related Coagulopathy: Is it A-O-K? Maybe Not, We Say. Pharm. Ther..

[90] Buitenhuis H.C., Soute B.A., Vermeer C. (1990). Comparison of the vitamins K1, K2 and K3 as cofactors for the hepatic vitamin K-dependent carboxylase. Biochim. Biophys. Acta.

[91] Azuma K., Ouchi Y., Inoue S. (2014). Vitamin K: Novel molecular mechanisms of action and its roles in osteoporosis. Geriatr. Gerontol. Int..

[92] Bouckaert J.H., Said A.H. (1960). Fracture healing by vitamin K. Nature.

[93] Hart J.P., Shearer M.J., Klenerman L., Catterall A., Reeve J., Sambrook P.N., Dodds R.A., Bitensky L., Chayen J. (1985). Electrochemical detection of depressed circulating levels of vitamin K1 in osteoporosis. J. Clin. Endocrinol. Metab..

[94] Booth S.L., Tucker K.L., Chen H., Hannan M.T., Gagnon D.R., Cupples L.A., Wilson P.W., Ordovas J., Schaefer E.J., Dawson-Hughes B. (2000). Dietary vitamin K intakes are associated with hip fracture but not with bone mineral density in elderly men and women. Am. J. Clin. Nutr..

[95] Kaneki M., Hodges S.J., Hosoi T., Fujiwara S., Lyons A., Crean S.J., Ishida N., Nakagawa M., Takechi M., Sano Y. (2001). Japanese fermented soybean food as the major determinant of the large geographic difference in circulating levels of vitamin K2: Possible implications for hip-fracture risk. Nutrition.

[96] Yaegashi Y., Onoda T., Tanno K., Kuribayashi T., Sakata K., Orimo H. (2008). Association of hip fracture incidence and intake of calcium, magnesium, vitamin D, and vitamin K. Eur. J. Epidemiol..

[97] Fusaro M., Cianciolo G., Brandi M.L., Ferrari S., Nickolas T.L., Tripepi G., Plebani M., Zaninotto M., Iervasi G., La Manna G. (2020). Vitamin K and Osteoporosis. Nutrients.

[98] Binkley N.C., Krueger D.C., Kawahara T.N., Engelke J.A., Chappell R.J., Suttie J.W. (2002). A high phylloquinone intake is required to achieve maximal osteocalcin gamma-carboxylation. Am. J. Clin. Nutr..

[99] Nakamura Y.S., Hakeda Y., Takakura N., Kameda T., Hamaguchi I., Miyamoto T., Kakudo S., Nakano T., Kumegawa M., Suda T. (1998). Tyro 3 receptor tyrosine kinase and its ligand, Gas6, stimulate the function of osteoclasts. Stem Cells.

[100] Yamaguchi M., Weitzmann M.N. (2011). Vitamin K2 stimulates osteoblastogenesis and suppresses osteoclastogenesis by suppressing NF-κB activation. Int. J. Mol. Med..

[101] Trivedi H.D., Danford C.J., Goyes D., Bonder A. (2020). Osteoporosis in Primary Biliary Cholangitis: Prevalence, Impact and Management Challenges. Clin. Exp. Gastroenterol..

[102] Orimo H., Nakamura T., Hosoi T., Iki M., Uenishi K., Endo N., Ohta H., Shiraki M., Sugimoto T., Suzuki T. (2012). Japanese 2011 guidelines for prevention and treatment of osteoporosis--executive summary. Arch. Osteoporos..

[103] Guanabens N., Cerdá D., Monegal A., Pons F., Caballería L., Peris P., Parés A. (2010). Low bone mass and severity of cholestasis affect fracture risk in patients with primary biliary cirrhosis. Gastroenterology.

[104] Stedman C.A., Liddle C., Coulter S.A., Sonoda J., Alvarez J.G., Moore D.D., Evans R.M., Downes M. (2005). Nuclear receptors constitutive androstane receptor and pregnane X receptor ameliorate cholestatic liver injury. Proc. Natl. Acad. Sci. USA.

[105] Wagner M., Halilbasic E., Marschall H.U. (2005). CAR and PXR agonists stimulate hepatic bile acid and bilirubin detoxification and elimination pathways in mice. Hepatology.

[106] Castano G., Burgueno A., Fernandez G.T., Pirola C.J., Sookoian S. (2010). The influence of common gene variants of the xenobiotic receptor (PXR) in genetic susceptibility to intrahepatic cholestasis of pregnancy. Aliment. Pharmacol. Ther..

[107] Karlsen T.H., Lie B.A., Frey F.K., Thorsby E., Broome U., Schrumpf E., Boberg K.M. (2006). Polymorphisms in the steroid and xenobiotic receptor gene influence survival in primary sclerosing cholangitis. Gastroenterology.

[108] Chen H.L., Liu Y.J., Chen H.L., Wu S.H., Ni Y.H., Ho M.C., Lai H.S., Hsu W.M., Hsu H.Y., Tseng H.C. (2008). Expression of hepatocyte transporters and nuclear receptors in children with early and late-stage biliary atresia. Pediatr. Res..

[109] Xie W., Radominska-Pandya A., Shi Y., Simon C.M., Nelson M.C., Ong E.S., Waxman D.J., Evans R.M. (2001). An essential role for nuclear receptors SXR/PXR in detoxification of cholestatic bile acids. Proc. Natl. Acad. Sci. USA.

[110] Li T., Chiang J.Y. (2013). Nuclear receptors in bile acid metabolism. Drug Metab. Rev..

[111] Bhalla S., Ozalp C., Fang S., Xiang L., Kemper J.K. (2004). Ligand-activated Pregnane X Receptor Interferes with HNF-4 Signaling by Targeting a Common Coactivator PGC-1α. Functional implications in hepatic cholesterol and glucose metabolism. J. Biol. Chem..

[112] Li T., Chiang J.Y. (2005). Mechanism of rifampicin and pregnane X receptor inhibition of human cholesterol 7α-hydroxylase gene transcription. Am. J. Physiol. Gastrointest. Liver Physiol..

[113] Halilbasic E., Baghdasaryan A., Trauner M. (2013). Nuclear receptors as drug targets in cholestatic liver diseases. Clin. Liver Dis..

[114] Wunsch E., Klak M., Wasik U., Milkiewicz M., Blatkiewicz M., Urasinska E., Barbier O., Bielicki D., Bogdanos D.P., Elias E. (2015). Liver Expression of Sulphotransferase 2A1 Enzyme Is Impaired in Patients with Primary Sclerosing Cholangitis: Lack of the Response to Enhanced Expression of PXR. J. Immunol. Res..

[115] Akita H., Suzuki H., Ito K., Kinoshita S., Sato N., Takikawa H., Sugiyama Y. (2001). Characterization of bile acid transport mediated by multidrug resistance associated protein 2 and bile salt export pump. Biochim. Biophys. Acta.

[116] Teng S., Piquette-Miller M. (2005). The involvement of the pregnane X receptor in hepatic gene regulation during inflammation in mice. J. Pharmacol. Exp. Ther..

[117] Kauffmann H.M., Keppler D., Gant T.W., Schrenk D. (1998). Induction of hepatic mrp2 (cmrp/cmoat) gene expression in nonhuman primates treated with rifampicin or tamoxifen. Arch. Toxicol..

[118] Courtois A., Payen L., Guillouzo A., Fardel O. (1999). Up-regulation of multidrug resistance-associated protein 2 (MRP2) expression in rat hepatocytes by dexamethasone. FEBS Lett..

[119] Fromm M.F., Kauffmann H.M., Fritz P., Burk O., Kroemer H.K., Warzok R.W., Eichelbaum M., Siegmund W., Schrenk D. (2000). The effect of rifampin treatment on intestinal expression of human MRP transporters. Am. J. Pathol..

[120] Teng S., Piquette-Miller M. (2007). Hepatoprotective role of PXR activation and MRP3 in cholic acid-induced cholestasis. Br. J. Pharmacol..

[121] Dempsey J.L., Wang D., Siginir G., Fei Q., Raftery D., Gu H., Cui Y.J. (2019). Pharmacological Activation of PXR and CAR Downregulates Distinct Bile Acid-Metabolizing Intestinal Bacteria and Alters Bile Acid Homeostasis. Toxicol. Sci..

[122] Wallace K., Cowie D.E., Konstantinou D.K., Hill S.J., Tjelle T.E., Axon A., Koruth M., White S.A., Carlsen H., Mann D.A. (2010). The PXR is a drug target for chronic inflammatory liver disease. J. Steroid Biochem. Mol. Biol..

[123] Ye N., Wang H., Hong J., Zhang T., Lin C., Meng C. (2016). PXR Mediated Protection against Liver Inflammation by Ginkgolide A in Tetrachloromethane Treated Mice. Biomol. Ther. (Seoul).

[124] Marek C.J., Tucker S.J., Konstantinou D.K. (2005). Pregnenolone-16alpha-carbonitrile inhibits rodent liver fibrogenesis via PXR (pregnane X receptor)-dependent and PXR-independent mechanisms. Biochem. J..

[125] Dai G., He L., Bu P. (2008). Pregnane X receptor is essential for normal progression of liver regeneration. Hepatology.

[126] Woolbright B.L. (2020). Inflammation: Cause or consequence of chronic cholestatic liver injury. Food Chem. Toxicol..

[127] Dai M., Hua H., Lin H., Xu G., Hu X., Li F., Gonzalez F.J., Liu A., Yang J. (2018). Targeted Metabolomics Reveals a Protective Role for Basal PPARα in Cholestasis Induced by α-Naphthylisothiocyanate. J. Proteome Res..

[128] Elsharkawy A.M., Mann D.A. (2007). Nuclear factor-kappaB and the hepatic inflammation-fibrosis-cancer axis. Hepatology.

[129] Miyoshi H., Rust C., Guicciardi M.E., Gores G.J. (2001). NF-kappaB is activated in cholestasis and functions to reduce liver injury. Am. J. Pathol..

[130] Racanelli V., Rehermann B. (2006). The liver as an immunological organ. Hepatology.

[131] El Kasmi K.C., Vue P.M., Anderson A.L., Devereaux M.W., Ghosh S., Balasubramaniyan N., Fillon S.A., Dahrenmoeller C., Allawzi A., Woods C. (2018). Macrophage-derived IL-1β/NF-κB signaling mediates parenteral nutrition-associated cholestasis. Nat. Commun..

[132] Lv P., Luo H.S., Zhou X.P., Xiao Y.J., Paul S.C., Si X.M., Zhou Y.H. (2007). Reversal effect of thalidomide on established hepatic cirrhosis in rats via inhibition of nuclear factor-kappaB/inhibitor of nuclear factor-kappaB pathway. Arch. Med. Res..

[133] Oakley F., Meso M., Iredale J.P., Green K., Marek C.J., Zhou X., May M.J., Millward-Sadler H., Wright M.C., Mann D.A. (2005). Inhibition of inhibitor of kappaB kinases stimulates hepatic stellate cell apoptosis and accelerated recovery from rat liver fibrosis. Gastroenterology.

[134] Wright M.C., Issa R., Smart D.E., Trim N., Murray G.I., Primrose J.N., Arthur M.J., Iredale J.P., Mann D.A. (2001). Gliotoxin stimulates the apoptosis of human and rat hepatic stellate cells and enhances the resolution of liver fibrosis in rats. Gastroenterology.

[135] Ozaki I., Zhang H., Mizuta T., Ide Y., Eguchi Y., Yasutake T., Sakamaki T., Pestell R.G., Yamamoto K. (2007). Menatetrenone, a vitamin K2 analogue, inhibits hepatocellular carcinoma cell growth by suppressing cyclin D1 expression through inhibition of nuclear factor kappaB activation. Clin. Cancer Res..

[136] Zhang H., Ozaki I., Hamajima H., Iwane S., Takahashi H., Kawaguchi Y., Eguchi Y., Yamamoto K., Mizuta T. (2011). Vitamin K2 augments 5-fluorouracil-induced growth inhibition of human hepatocellular carcinoma cells by inhibiting NF-κB activation. Oncol. Rep..

